# Can the Inclusion of a Vegetable Biocholine Additive in Pig Feed Contaminated with Aflatoxin Reduce Toxicological Impacts on Animal Health and Performance?

**DOI:** 10.3390/ani13193010

**Published:** 2023-09-25

**Authors:** Vanessa Dazuk, Lara Tarasconi, Vitor Luiz Molosse, Bruno Giorgio Oliveira Cécere, Guilherme Luiz Deolindo, João Vitor Strapazzon, Nathieli Bianchi Bottari, Bianca Fagan Bissacotti, Maria Rosa Chitolina Schetinger, Laércio Sareta, Ricardo Evandro Mendes, Marcelo Vedovatto, Eduardo Micotti Gloria, Diovani Paiano, Gabriela Miotto Galli, Aleksandro Schafer Da Silva

**Affiliations:** 1 Programa de Pós-graduação em Zootecnia, Universidade do Estado de Santa Catarina (UDESC), Chapecó 89815-630, Brazil; 2 Departamento de Zootecnia, Universidade do Estado de Santa Catarina (UDESC), Chapecó 89815-630, Brazil; 3 Departamento de Bioquímica e Biologia Molecular, Universidade Federal de Santa Maria (UFSM), Santa Maria 97105-900, Brazil; 4 Laboratório de Patologia Veterinária, Instituto Federal Catarinense, Concordia 89051-000, Brazil; 5 Athens Veterinary Diagnostic Laboratory (AVDL), Department of Pathology, College of Veterinary Medicine, University of Georgia (UGA), Athens, GA 30602, USA; 6 Dean Lee Research and Extension Center, Louisiana State University, Alexandria, LA 71302, USA; 7 Departamento de Agroindústria, Alimentos e Nutrição, Escola Superior de Agricultura Luiz de Queiroz, Universidade de São Paulo, São Paulo 05508-090, Brazil

**Keywords:** supplementation, mycotoxins, nutrition, swine, toxicology

## Abstract

**Simple Summary:**

Our goal was to determine whether adding vegetable biocholine (VB) to pigs’ diets would minimize the negative effects caused by daily aflatoxin (B1 + B2) intake. The consumption of feed contaminated by aflatoxin reduced feed consumption and weight gain in piglets. Intake of aflatoxin in the diet also can cause subclinical intestinal and hepatic oxidative stress. VB supplementation in piglet diets had no positive effects on performance. VB showed hepaprotective potential in the face of a challenge with aflatoxin.

**Abstract:**

(1) Background: This study’s objective was to determine whether adding vegetable biocholine (VB) to pigs’ diets would minimize the negative effects caused by daily aflatoxin (B1 + B2) intake. (2) Methods: We used seventy-two whole male pigs weaned at an average of 26 days and divided them into four groups with six replicates each (2 × 2 factorial). The treatments were identified as Afla0VB0 (negative control, without aflatoxin and without VB); Afla500VB0 (positive control, 500 µg/kg of aflatoxins; Afla0VB800 (800 mg/kg of VB); and Afla500VB800 (500 µg/kg of aflatoxin +800 mg/kg of VB). (3) Results: In the first 20 days of the experiment, only the pigs from Afla500VB0 had less weight gain and less feed consumption, different from the 30th to 40th day, when all treatments had lower performance than the negative control. In the liver, higher levels of oxygen-reactive species and lipid peroxidation were observed in Afla500VB0, associated with greater activity of the enzymes alanine aminotransferase and aspartate aminotransferase. In the jejunum, oxidative stress was associated with nitrous stress in Afla500VB0. An increase in splenic glutathione S-transferase activity in the Afla500VB800 animals was observed. (4) Conclusions: Consuming a diet contaminated with 500 µg/kg of aflatoxin influences the health and performance in the nursing phase in a silent way; however, it generates high economic losses for producers. When VB was added to the pigs’ diet in the face of an aflatoxin challenge, it showed hepatoprotective potential.

## 1. Introduction

The nursery phase of swine production systems is challenging and is critically important to subsequent phases of the production cycle [1]. The weaning of piglets is considered critical and requires specific care because of various challenges and changes that the animals are subjected to simultaneously. The replacement of liquid feed (sow milk) for solid feed based on ingredients of plant origin such as corn or soybean meal exposes the animals to mycotoxins found in dietary cereals [2]. The result is often increased mortality rates and delayed performance [3].

Mycotoxins in animal feeds are a constant concern regarding animal protein, eggs, and milk due to economic losses [4]. According to Dilkin [5], aflatoxins are present in approximately 38% of pig diets and are responsible for the most significant swine mycotoxicoses; they represent a severe condition for swine health. Mycotoxin effects can vary; however, they generally depress the immune system [6,7], resulting in slower weight gain, digestive disorders, and liver disease [8]. Immunotoxicity is also reported in pigs subjected to feed contaminated with aflatoxin B1 and fumonisin B1 and these mycotoxins exert their toxic effects via various biochemical mechanisms [9]. The most significant problem surrounding mycotoxicosis derives from losses related to the functionality of organs and systems of animals, implying a decrease in their productive performance [5]. The liver is most affected by the toxic effects of aflatoxin, resulting in a series of changes in the metabolism of proteins, carbohydrates, and lipids [10], in addition to causing clinically apparent hepatic toxicosis [11].

For all these reasons, it is critical to search for ingredients or additives that, when added to animal feed, minimize the effects of the consumption of mycotoxins. In this sense, natural products derived from plants may help protect animals from toxic agents derived from feed and the environment that damage health and performance. Vegetable biocholine (VB) in fish diet improved health and performance [12]; when these fish were challenged with aflatoxin B1, biocholine had a hepatoprotective effect [12]. A similar effect was observed in laying hens exposed to aflatoxin B1 [13]. Based on this information, we aimed to determine whether adding VB to pigs’ diet would minimize the adverse effects caused by daily aflatoxin intake, focusing on performance and health.

## 2. Materials and Methods

### 2.1. Product VB

We used commercially available VB (Biocholine Powder^®^, Technofeed, Sao Paulo, Brazil). The product is produced from plant extracts (*Trachyspermum ammi*, *Azadichara indica,* and *Achyranthes rugas*). The colorimetry method was used to determine tannins, phenolics, and flavonoids: Brazilian Compendium of Animal Feed, 2013, method 52. A sample of this additive was used for the quantification of choline in VB using high-performance thin-layer chromatography, as described by Kupke and Zeugner [14]. The amount of tannins was measured by colorimetry, following method 52, *Compêndio Brasileiro de aliementação Animal*, 2013; and that of flavonoids was measured following the methodology described by Dazuk et al. [13]. We used 800 mg VB/kg of feed provided to pigs, based on the results of Souza et al. [12].

### 2.2. Aflatoxin Production and Analysis

Aflatoxins were produced by the ATCC 13608 strain of *Aspergillus flavus* during fermentation of converted rice, and the following protocol was used. We used 500 mL Erlenmeyer flasks in which we placed 100 g of rice. At least 2 h before the sterilization, 40 mL of distilled water was added to flask and mixed with the rice. The sterilization was performed at 121 °C for 30 min, and then the flasks were left to cool before inoculation. The rice was inoculated with 2 mL of 10^8^ *A. flavus* spores mL^−1^. The incubation was carried out for 21 days at a controlled temperature (250 °C) and with constant stirring of flasks. After incubation, the fermented material was dried in an oven at 50 ºC and was ground. The concentration of aflatoxin in the inoculum was determined in advance (result: 796 mg of aflatoxin B1 + B2/kg of rice) to calculate and determine the amount added in the diets in order to obtain a 500 µg of aflatoxin B1 + B2/kg contamination, a dose that delays the growth of pigs [6].

Samples of feed and inoculum were ground to <0.85 mm in size, and 1 g of the ground material was transferred to a 50 mL test tube. We added 10 mL of ultrapure water and 10 mL of acetonitrile/acetic acid (CH_3_CN:CH_3_COOH) (99.5:0.5, *v*/*v*), and the test tube was placed in a mechanical shaker for 10 min. A mixture of 4 g of MgSO_4_ and 1 g of NaCl was added, and the tube was vigorously hand-shaken for 10 s. The solution was centrifuged for 15 min at 5000× *g*, at 25 °C, and 2.5 mL of supernatant was transferred to a capped glass test tube where 2.5 mL of hexane was added. The solution was shaken for 2 h and then centrifuged at 1000× *g*, at 20 °C for 1 min. From the lower phase (acetonitrile), 1 mL was withdrawn and dried with a nitrogen stream at 40 °C. The reconstitution was performed with 75 µL of methanol in an ultrasonic bath for 10 s, then 10 s in a test tube mixer after adding 75 µL of ultrapure water. After centrifugation for 10 min at 14,000× *g*, 60 µL was withdrawn and transferred to a vial where 140 µL of ultrapure water was added. Ten microliters were injected into the chromatographic system.

Detection and quantification of aflatoxins were performed using high-performance liquid chromatography coupled with tandem mass spectrometry (LC/MS/MS). Chromatographic separation was carried out using an Acquity UPLC System (Waters, Milford, MA, USA) equipped with a 100 × 2.1 mm, 1.7 µm Acquity UPLC BEH C18 column (Waters, Milford, MA, USA). The column was maintained at 40 °C, and the injection volume was 10 µL. The mobile phase consisted of 0.1% formic acid in water (A) and 0.1% formic acid in acetonitrile (B). The acetonitrile (B) concentration was raised gradually from 10% to 90% within 12 min, brought back to the initial conditions after 0.1 min, and allowed to stabilize for 3 min. The mobile phase was delivered at a 0.4 mL/min flow rate. The LC system was coupled with a Xevo TQS tandem mass spectrometer (Waters, Milford, MA, USA), equipped with a turbo-ion electrospray ion (ESI) source. In positive mode, the mass spectrometer was operated in scheduled multiple reaction monitoring (MRM). The electrospray ionization and MS/MS conditions are shown in Table A1.

### 2.3. Animals and Experimental Design

The experiment was carried out in the experimental pig house at the Experimental Farm of the State University of Santa Catarina (FECEO), located in Guatambu, SC, Brazil, over 40 days. The composition of the diet is described in Table 1.

We used 72 uncastrated male piglets (7.42 ± 1.27 kg) weaned at an average of 26 days, divided into 4 groups with 6 replicates each and 3 pigs per repetition. The experiment was conducted in a nursery facility with a plastic floor suitable for the phase, troughs with the availability of 15 cm trough/animal, and automatic type drinking troughs with a flow rate of 1 L/min. The installation was heated using an electric heater. The treatments were as follows: Afla0VB0, negative control (without aflatoxin and without VB); Afla500VB0, positive control (500 µg/kg of aflatoxin); Afla0VB800, 800 mg/kg of VB; and Afla500VB800, 500 µg/kg of aflatoxin + 800 mg/kg of VB.

The values were calculated based on the feed composition proposed by Rostagno et al. [15] and the nutritional composition of the base mix.

### 2.4. Growth Performance

The experimental period included evaluation of growth performance on days 10, 20, 30, and 41. During these periods, individual pigs and leftover feed were weighed using an electronic scale (model DIGI-TRON UL-5 with column). The rations were stored in individual buckets, one for each repetition. Daily weight gain (DWG) and daily feed intake (DFI) were measured, from which feed conversion ratio (FCR) was obtained. Daily feed consumption was measured by weighing the feed provided at the beginning of each period and leftovers at the end of each stage and weighing the pigs at that time for the DWG. The FC data were calculated as feed consumption/weight gain.

### 2.5. Sample Collection

Blood samples were collected through the cranial vena cava in vacutainer tubes on days 0, 10, 20, 30, and 40 of the experimental period in tubes containing anticoagulant ethylenediaminetetraacetic acid (EDTA). First, complete blood counts were performed according to the methodology described below, and a 0.5 mL aliquot of blood was removed for analysis of CAT and SOD activity and stored frozen. Subsequently, blood was centrifuged at 2350× *g* for 5 min, obtaining serum which was contained in a microtube and maintained frozen (−20 °C) until biochemical analysis.

On day 32 of the experiment, six animals from each group were slaughtered in a specialized slaughterhouse through electrical stunning, according to the current legislation of the inspection system. The liver, intestine (jejunum), and spleen samples were collected, and samples were preserved in 10% formaldehyde. A liver sample was homogenized in saline, centrifuged (2800× *g* for 10 min), and the supernatant was removed. These were packed in microtubes and frozen for further analysis of oxidants/antioxidants.

### 2.6. Hemogram

The manufacturers’ recommendations determined the hemoglobin concentrations and total leukocyte and erythrocyte counts using commercial kits. Blood smears were made and stained with commercial dye (Rapid Panotype) to perform differential leukocyte counts under a light microscope with a 1000× *g* magnification, as described by Lucas and Jamroz [16]. Hematocrit was measured using microcapillary tubes and centrifuged at 14,000× *g* for 5 min.

### 2.7. Serum Biochemical Indices

The serum concentration of total proteins, albumin, cholesterol, triglycerides, alanine aminotransferase (ALT), and aspartate aminotransferase (AST) was measured using semi-automatic BioPlus equipment (Bio-2000, Bioplus Produtos para Laboratórios, Ltda, Barueri, SP, Brazil) and specific commercial kits. Serum globulin concentration was calculated as the difference between serum levels of total proteins and albumin.

### 2.8. Oxidizing and Antioxidant Status

Serum activity of glutathione S-transferase (GST) and blood activities of superoxide dismutase (SOD) and catalase (CAT) were measured. With modifications, GST activity was measured according to Mannervik and Guthenberg [17]. Briefly, GST activity was measured as the formation rate of dinitrophenyl-S-glutathione at 340 nm in a medium containing 50 mM potassium phosphate with pH 6.5, 1 mM GSH, 1 mM 1-chloro-2, 4-dinitrobenzene (CDNB) as substrate and tissue supernatants (approximately 0.8–1.0 mg protein). The results were expressed as U GST/mg protein. The activity of the SOD was measured using the method of Marklund and Marklund [18], and the results were expressed as nmol SOD/mg of protein. CAT activity was measured using ultraviolet spectrometry, according to the method of Aebi [19], and the results were expressed as nmol CAT/mg of protein.

The concentration of reactive oxygen species (ROS) in serum was analyzed by the method described by Halliwell and Gutteridge [20]. The serum (10 μL) was incubated with 12 μL of dichlorofluorescein (DFC) per mL at 37 °C for 1 h in the dark. Fluorescence was determined using 488 nm for excitation and 520 nm for emission. The results were expressed as UDCF/mg protein. NOx levels were measured according to the method of Miranda et al. [21], which indirectly quantifies nitrite/nitrate levels, and the results were expressed as U NOx/mg protein. TBARS values were obtained using the method described by Ohkawa et al. [22] in tissues and Jentzsch et al. [23] in the plasma and expressed as nmol MDA/mL.

### 2.9. Organ Weight and Histopathology

Spleen and liver were weighed during the slaughter process. Then, samples of the liver, jejunum, and spleen were preserved in a formaldehyde solution (10%). Tissue samples were processed and placed in paraffin blocks. Then, sections were made and stained with hematoxylin–eosin (HE).

### 2.10. Statistical Analyses

The experimental design of this study was one factorial 2 × 2 (feed with and without aflatoxin (Afla0 and Afla500) and with (VB0) and without VB (VB800)). All variables were subjected to the normality test (Shapiro–Wilk). All data were analyzed using the MIXED procedure of SAS (SAS Inst. Inc., Cary, NC, USA; version 9.4), with Satterthwaite approximation to determine the denominator’s degree of freedom for the test of fixed effects. DWG, DFI, and FC data were tested for fixed effects of aflatoxin, VB, and the interaction, and as a random effect, we included pen (aflatoxin × VB). The data of antioxidant response in liver, spleen, and jejunum were tested for fixed effects of aflatoxin, VB, and the interaction, and random effects included pen (aflatoxin × VB) and animal (pen). All other data were analyzed as repeated measures (body weight and blood variables), aflatoxin, VB, day, and all possible interactions were included as fixed effects, and the random effects included pen (aflatoxin × VB) and animal (pen). The compound symmetric covariance structure was selected according to the lowest Akaike information criterion. Means were separated using PDIFF, and all results were reported as LSMEANS followed by SEM. A simple Pearson correlation was evaluated among the antioxidant variables using the CORR procedure of SAS to determine the interrelation between them. Significance was defined when *p* ≤ 0.05 and tendency when *p* > 0.05 and ≤0.10.

## 3. Results

### 3.1. Biocholine

The product had the following chemical composition: 961 g/kg of dry matter; 858 g/kg of organic material; 45 g/kg protein; 440 g/kg neutral detergent fiber; 381 g/kg acid detergent fiber; 57.9 g/kg ether extract; 15.7 g/kg calcium; and 3.08 g/kg phosphorus. Analysis of the VB identified 83.4% of the composition of the commercial product, of which 50.9% was total choline (12.5% phosphatidylethanolamine, 23.2% phosphatidylinositol, 46.4% phosphatidylcholine, 8.9% lyso phosphatidylcholine, and 16.1% phosphatidylcholine “natural choline conjugates”), 10.8 g/kg total tannins, and 2.03 g/kg total flavonoids.

### 3.2. Growth Performance

**Figure 1:**
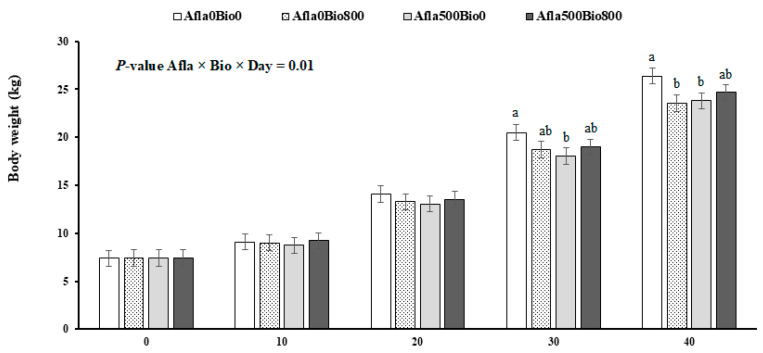
Growth of pigs fed with diets containing aflatoxins (Afla) and VB. In a factorial design (2 × 2) included no aflatoxin or aflatoxin (Afla0 and Afla500 with 0 or 500 µg/kg of aflatoxin/kg of concentrate, respectively) and also included no VB or VB (VB0 and VB800 with 0 or 800 mg of VB/kg of concentrate, respectively). ^a,b^ Differences (*p* ≤ 0.05) between treatments. Vertical bars represent the SEM.

The performance results are presented in Table 2. There were significant interactions between treatments (AFLA versus VB) in the first 30 days of the experiment; i.e., from days 1 to 10 (*p* ≤ 0.01) and from day 1 to 20 (*p* ≤ 0.05), the positive control group (Afla500VB0) had significantly lower DWG than did the negative control group (Afla0VB0). From 1 to 30 days, the negative control (Afla0VB0) had more significant weight gain than the other groups (*p* ≤ 0.03). The average body weights of the four groups are shown in Figure 1. In general, the positive control group (Afla500VB0) had lower body weights at 30 and 41 days compared to Afla0VB0, with a positive effect on those challenged and supplemented with VB (Afla500VB800). From days 1 to 30, the highest feed consumption was in Afla0VB0 compared to the others. FCR did not differ significantly between treatments (*p* > 0.05).

### 3.3. Serum Biochemical Indices

Protein and lipid metabolism and liver enzyme activities are presented in Table 3. We found an interaction between day and aflatoxin intake and an effect of day and VB in pigs’ serum. In general, the inclusion of aflatoxin in the diet increased (*p* ≤ 0.01) levels of ALT and AST on days 20, 30, and 40 when compared to the other treatments (Table 3). No significant differences were found in comparing groups with or without aflatoxin in any of the evaluated periods (*p* > 0.05) for total protein, albumin, and globulin. However, when comparing the groups with and without VB, we found lower levels of total proteins and globulins in the VB800 group on day 10 (*p* ≤ 0.01). Cholesterol levels were not significantly different among any of the compared groups. On day 10, triglyceride levels were lower in the groups with aflatoxin (Afla500VB0) and with aflatoxin and VB (Afla500VB800) (*p* ≤ 0.01).

### 3.4. Hemogram

Complete blood count results are displayed in Table 4. Counts of eosinophils, lymphocytes, leukocytes, and erythrocytes did not differ significantly among groups (*p* > 0.05). Regarding hematocrit, there was an effect of day on pigs that consumed aflatoxin; that is, on days 20 and 30, the hematocrits were higher in pigs of groups Afla500VB0 and Afla500VB800 (*p* ≤ 0.01). Pigs in the Afla500 groups on the 20th and 30th days had lower neutrophil counts than did the Afla0 groups (*p* ≤ 0.05). On day 10, monocyte counts were lower in the groups of pigs that consumed aflatoxin (Afla500VB0 and Afla500VB800—*p* ≤ 0.01) on the 20th day. Pigs in the Afla500VB800 group had higher monocyte counts on day 40 than did the Afla0VB800 group (*p* ≤ 0.01).

### 3.5. Serum, Blood, and Tissue Antioxidant Responses

Serum and whole blood antioxidant responses are shown in Table 5. There were no significant differences between the groups concerning GST, SOD, CAT, NOx, ROS, or TBARS (*p* > 0.05). The results of the oxidative and antioxidant status in tissues are shown in Table 6. In the liver, NOx levels were higher in the Afla0VB0 and Afla500VB800 groups than in the others (*p* ≤ 0.01); also, in the liver, the levels of ROS (*p* ≤ 0.01) and TBARS (*p* ≤ 0.03) were higher in pigs in the Afla500VC0 group. In the spleen, ROS levels were lower in the Afla500VB0 group (*p* ≤ 0.05). TBARS levels were lower in Afla500VB800. In the spleen, there was also an effect of the consumption of VB on GST activity; that is, more significant activity was observed in the animals of the Afla500VB800 group (*p* ≤ 0.01). In the jejunum, an effect of aflatoxin consumption was also observed; that is, intake of aflatoxin increased the activity of GST, as well as that of NOx and ROS (Table 6) (*p* ≤ 0.01). Table 7 shows Pearson correlation coefficients among antioxidant variables in the blood, liver, spleen, and jejunum. Significant correlations were observed in the liver and blood between the following variables: TBARS versus GST, ROS versus ROS, and GST versus NOx. In the blood and spleen, the following were significant correlations: ROS versus TBARS and GST versus TBARS (*p* ≤ 0.05); in the blood and jejunum, significant correlations were found for TBARS versus TBARS *p* ≤ 0.05); in the liver and spleen, significant correlations were found for NOx versus ROS (*p* ≤ 0.05); in the liver, significant correlations were found for TBARS versus ROS (*p* ≤ 0.05); in liver and jejunum, significant correlations were found for TBARS versus NOx and ROS versus NOx/ROS/GST (*p* ≤ 0.05); in the spleen and jejunum, significant correlations were found for GST versus TBARS (*p* ≤ 0.05); in the jejunum, significant correlations were found for NOx versus ROS/GST (*p* ≤ 0.05).

### 3.6. Carcass Yield and Liver and Spleen Weight

Pigs that consumed VB in the diet had higher carcass yields than the control (Table 8). Liver weights were higher in positive control pigs; that is, animals fed with aflatoxin (Table 8); however, VB supplementation in the diet of pigs challenged with mycotoxin minimized this change (*p* > 0.05). The spleen weight was higher in pigs supplemented only with VB (Table 8).

### 3.7. Histopathology

**Figure 2:**
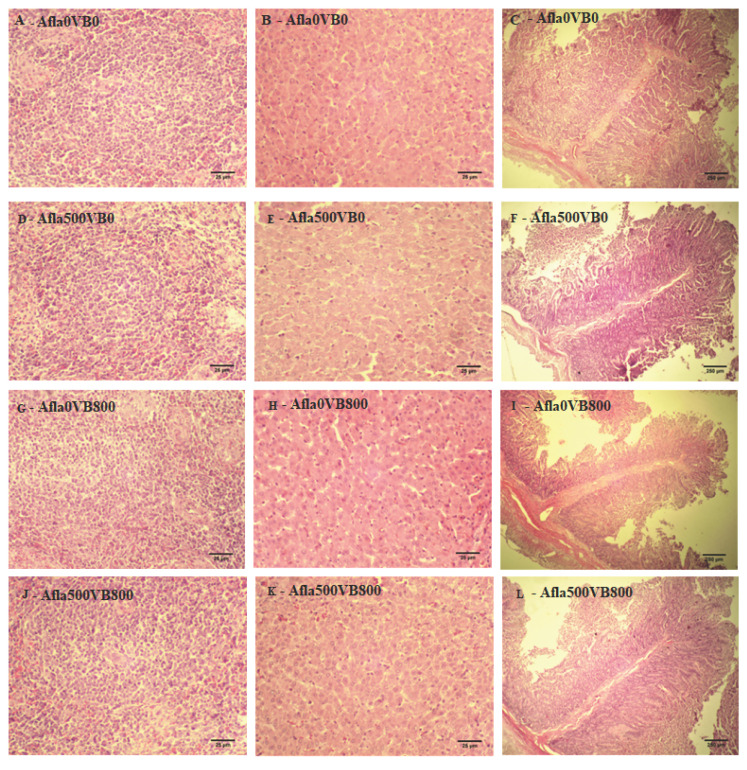
No lesions were observed in the spleen (**A**,**D**,**G**,**J**), liver (**B**,**E**,**H**,**K**), and jejunum (**C**,**F**,**I**,**L**) of animals.

No intestinal, hepatic, or spleen lesions were observed in any treatment (Figure 2). The intestinal fold size was smaller in the pigs of the Afla500VB0 and Afla0VB800 groups compared to the others (*p* ≤ 0.05) (Table 8). Piglets that consumed aflatoxin (Afla500VB0) had higher villous height, and Afla500VB0 and Afla500VB800 had a greater depth of intestinal crypts than the others (*p* ≤ 0.05) (Table 8).

### 3.8. Mycotoxin Analysis in Feed

After the analyses described in Section 2.2, we determined that the aflatoxins contaminating the diets were as follows: Afla0Bio0 (AFLAB1 = 0.0 µg/kg; AFLAB2 = 0.0 µg/kg); Afla500VB0 (AFLAB1 = 471.8 µg/kg; AFLAB2 = 8.2 µg/kg); Afla0VB800 (AFLAB1 = 0.0 µg/kg; AFLAB2 = 0.0 µg/kg); Afla500VB800 (AFLAB1 = 335.1 µg/kg; AFLAB2 = 6.4 µg/kg). AFLAG1 and AFLAG2 were not observed in the experimental feed.

## 4. Discussion

The aim of this study was to determine whether adding VB to pigs’ diet would minimize the adverse effects caused by daily aflatoxin intake, focusing on performance and health. In general, we observed lower weight gain in pigs that consumed aflatoxin in the diet, and there were also changes in intestinal morphometry. Consumption of feed with VB at 800 mg/kg harmed weight gain. The negative effect of aflatoxin was expected because, according to the literature, the sensitivity of pigs to this mycotoxin is the most substantial among animal species [10]. Most aflatoxicosis in swine production is silent, as a noticeable clinical picture is not frequently seen, although losses in weight gain have been observed, as was observed in the present study. In a study with diets contaminated with 500 µg/kg of aflatoxin supplied to weaned pigs, researchers found a reduction in the growth rate of the animals [14], a result similar to those described by Santurio [10]. In the first 20 days of the nursing phase, VB prevented adverse effects of aflatoxicosis on the body weight and feed consumption (Afla500VB800) because the weight gain was similar to that of the animals that did not consume the mycotoxin in the diet (Afla0VB0). However, between days 21 to 30, weight gain was lower in the animals of all treatments compared to the control, suggesting that supplementation with VB did not prevent the adverse effects caused by aflatoxin and even interfered with pigs’ development when used only as an additive (Afla0VB800). We believe that the dose of 800 mg VB/kg of feed was high and was responsible for the negative effect on weight gain. A study conducted in the 1980s concluded that the supplementation of pigs with choline via the diet should avoid excesses when it is desired to obtain maximum performance gains [24]. Similarly, in the 1980s, researchers reported reduced gain and efficiency in broilers fed with a choline level only slightly higher than the requirement [25]. As VB contains a choline source known as phosphatidylcholine, which needs to be better studied in pigs and as our study shows that the dose used was not correct, in future studies, it will be necessary to test lower doses in order to calculate the ideal dose capable of enhancing performance, as described in other animal species [26,27,28].

The effects of aflatoxin were manifested as higher serum activities of ALT and AST in piglets that had aflatoxin and did not consume VB; however, histologically, no changes were observed. The liver is the main organ damaged by aflatoxin [5,7,10,29,30]. High levels of ALT and AST indicate liver damage, and their values can be elevated in the context of fungal and bacterial infections [31]. Triglyceride levels were lower in the serum of piglets that received the combination of aflatoxin and biocholine compared to the negative control; however, there was no conclusive explanation for this change. We know that the synthesis of fats occurs in adipose tissue and the liver; triglycerides are secreted in the bloodstream for use in other tissues [32]; furthermore, animals affected by aflatoxicosis undergo essential changes in hepatic metabolism that affects fat metabolism [33]; this may explain the lower values of triglycerides in the group of animals subjected to aflatoxin.

There was an effect of aflatoxin on hematocrit; a lower percentage of hematocrit was observed in pigs that consumed mycotoxin. This result differed from that of Muller et al. [34], who fed pigs a diet contaminated experimentally with aflatoxin and fumonisin. These authors reported greater hematocrit and hemoglobin concentrations. On days 20 and 40 of the experiment, we recorded higher neutrophil counts and lower monocyte counts in the blood of piglets that consumed aflatoxin (positive control) compared to the others, suggesting that the intake of VB prevented these alterations. According to Murphy [35], neutrophils are the body’s first line of defense; they act by phagocytosing fungi, bacteria, and dead tissue during inflammatory processes and injuries resulting from aflatoxicosis. In another study by our research group, where piglets were challenged with diets contaminated with aflatoxin and fumonisin, we found a reduction in total leukocyte counts; however, there was no change in the neutrophil counts [36].

Variables indicative of nitrous stress (NOx) and oxidative stress (ROS and TBARS) were elevated in the jejunum of pigs in the group exposed to aflatoxin; furthermore, in the livers of these animals, there was a higher concentration of ROS and TBARS associated with a larger size of the liver. Lower ROS levels were also found in the spleen. The formation of free radicals by the body under normal conditions is inevitable as they are necessary for the cellular respiration process; however, the production of ROS is higher in animals when tissue injuries are caused by trauma, infections, parasites, toxins, and extreme exercise. In the spleen, lower levels of TBARS and more significant GST activity in piglets of the Afla500Bio800 group are positive findings in terms of animal health and may be related to the known antioxidant effect of VB [27]; nevertheless, this effect was modest in our study. When VB was used in diets of Nile tilapia challenged with aflatoxin B1 [11], the results of the antioxidant effect were substantial, unlike what we saw in the present study, where no serum alteration in the oxidant/antioxidant status was found; we believe this is related to the subclinical and silent toxicity of this mycotoxin.

We observed correlations among antioxidant variables in blood, liver, spleen, and jejunum in various tissues. This suggests aflatoxin-mediated interference in the functioning of the organs, leading to damage to the health of animals that consume it, as described by other authors [6,27,30,36,37,38]. Elevated oxidative biomarkers indicate cell and tissue damage, which is probably related to aflatoxin consumption [11]; an important diagnostic tool for alterations caused by mycotoxicosis, since histologically lesions were not observed.

## 5. Conclusions

The consumption of feed contaminated by AFLB1 reduced feed consumption and weight gain in pigs; it also caused subclinical intestinal and hepatic oxidative stress, in addition to increasing the activity of liver enzymes that are biomarkers of liver damage. VB intake by pigs in the diet had no positive effects on performance; however, it minimized the adverse effects of the feed contaminated by aflatoxin B1 in the first 20 days of the nursing phase. Antioxidant responses to VB were not seen; nevertheless, we believe such responses occurred, and the additive probably prevented exacerbated, undesirable oxidative reactions in the animals by increasing levels of free radicals and tissue lipid peroxidation. VB demonstrates a possible hepatoprotective effect, which deserves further research in order to confirm and understand the mechanisms.

## Figures and Tables

**Table 1:** Ingredients and nutritional composition of diets.

Items, (g/kg as Feed Base)	Pre-Initial I	Pre-Initial II	Initial I
Ground corn, 7.8% CP	400	500	650
Soybean meal, 46% CP	100	250	300
Pre-initial base mix I ^1^	500	-	-
Pre-initial base mix II ^2^	-	250	-
Initial base mix I ^3^	-	-	50
Calculated composition, (as feed base)			
Crude protein (g/kg)	202	203	199
Metabolizable energy (Mcal/kg)	3.52	3.43	3.36
Calcium (g/kg)	6.80	7.02	7.01
Available phosphorus (g/kg)	3.39	3.56	3.38
Digestible lysine (g/kg)	14.5	13.5	12.8
Digestible methionine (g/kg)	5.85	5.14	4.78
Digestible threonine (g/kg)	1.16	1.08	1.02

^1^ Base mix minimum levels by kg as feed base: crude protein (CP) 210 g, crude fat 55 g, calcium 10 g, total phosphorus 7 g, sodium 6 g, Co 1.6 mg, Cu 300 mg, Fe 300 mg, I 3.6 mg, Mn 110 mg, If 0.8 mg, Zn 5.4 g, Cr 0.6 mg, vit. A 29,000 IU, vit. D3 6000 IU, vit. E 160 IU, vit. K3 7 mg, vit. B1 7 mg, vit. B2 11 mg, vit. B6 7 mg, vit. B12 140 µg, folic acid 1.4 mg, nicotinic acid 81 mg, pantothenic acid 51 mg, choline 1.9 g, biotin 0.2 mg, lysine 22 g, methionine 8000 mg, phytase 1000 FTU, xylanase 3000 EPU, *S. cerevisiae* 4.8 × 10^9^, *L. acidophilus* 5.5 × 10^7^, *B. bifidum* 3.9 × 10^7^, *B. amyloliquefaciens* 1.2 × 10^8^. The maximum levels by kg as feed base are: humidity 90 g, crude fiber 20 g, Ca 14 g, ash 400 g. ^2^ Base mix minimum levels by kg as feed base: crude protein 160 g, crude fat 50 g, calcium 18 g, phosphorus 10 g, sodium 10 g, Co 3.2 mg, Cu 600 mg, Fe 600 mg, I 7.2 mg, Mn 220 mg, If 1.6 mg, Zn 10 g, Cr 1.2 mg, vit. A 58,000 IU, vit. D3 12,000 IU, vit. E 320 IU, vit. K3 14 mg, vit. B1 14 mg, vit. B2 23 mg, vit. B6 14 mg, vit. B12 280 mcg, folic acid (min) 2.8 mg, nicotinic acid (min) 163 mg, pantothenic acid 102 mg, choline 2120 mg, biotin 0.4 mg, lysine 25 g, methionine 10 g, phytase 2000 FTU, xylanase 6000 EPU. Maximum levels per kg as feed base: humidity 50 g, crude fiber 30 g, Ca 25 g, ashes 450 g. ^3^ Base mix minimum levels by kg as feed base: base mix minimum levels per kg as feed base for the second formulation are: calcium 90 g, phosphorus 20 g, sodium 35 g, Cu 1000 mg, Fe 1000 mg, I 20 mg, Mn 500 mg, If 8 mg, Zn 15 g, vit. A 180,000 IU, vit. D3 36,000 IU, vit. E 400 IU, vit. K3 60 mg, vit. B1 28 mg, vit. B2 80 mg, vit. B6 30 mg, vit. B12 360 mcg, folic acid 8 mg, nicotinic acid 600 mg, pantothenic acid 320 mg, choline 3120 mg, biotin 2 mg, lysine 40 g, methionine 40 g, threonine 5500 mg, zinc bacitracin 900 mg. Maximum levels per kg as feed base: humidity 20 g, ashes 730 g, Ca 160 g.

**Table 2:** Performance of pigs fed with diets containing aflatoxins and biocholine (VB).

	Treatments ^2^				SEM	*p*-Values ^3^		
Variables ^1^	Afla0VB0	Afla500VB0	Afla0VB800	Afla500VB800		Afla × VB	Afla	VB
DWG, kg								
d 1 to 10	0.172 ^a^	0.130 ^b^	0.156 ^ab^	0.180 ^a^	0.01	<0.01	0.40	0.16
d 1 to 20	0.335 ^a^	0.281 ^b^	0.293 ^ab^	0.306 ^ab^	0.01	0.05	0.29	0.64
d 1 to 30	0.410 ^a^	0.332 ^b^	0.353 ^b^	0.360 ^b^	0.02	0.03	0.05	0.43
d 11 to 20	0.497	0.432	0.430	0.431	0.03	0.28	0.30	0.28
d 21 to 30	0.536 ^a^	0.416 ^b^	0.453 ^b^	0.451 ^b^	0.02	0.02	0.01	0.30
d 31 to 41	0.651	0.639	0.535	0.633	0.04	0.21	0.32	0.17
d 1 to 41	0.468 ^a^	0.397 ^b^	0.392 ^b^	0.423 ^ab^	0.03	0.01	0.01	0.12
DFI, kg								
d 1 to 10	0.295 ^a^	0.245 ^b^	0.258 ^ab^	0.293 ^a^	0.02	0.04	0.71	0.77
d 1 to 20	0.465	0.408	0.412	0.437	0.03	0.14	0.56	0.65
d 1 to 30	0.662 ^a^	0.583 ^b^	0.553 ^b^	0.580 ^b^	0.04	0.05	0.22	0.39
d 11 to 20	0.633	0.572	0.558	0.583	0.04	0.27	0.63	0.41
d 21 to 30	0.998 ^a^	0.760 ^b^	0.795 ^b^	0.815 ^b^	0.07	0.05	0.13	0.30
d 31 to 41	1.181 ^a^	1.050 ^b^	1.017 ^b^	1.072 ^ab^	0.06	0.07	0.49	0.21
d 1 to 41	0.801 ^a^	0.708 ^b^	0.677 ^b^	0.712 ^b^	0.05	0.05	0.12	0.36
FCR								
d 1 to 10	1.703	1.915	1.683	1.645	0.10	0.23	0.40	0.17
d 1 to 20	1.383	1.472	1.402	1.437	0.06	0.63	0.28	0.88
d 1 to 30	1.605	1.627	1.570	1.611	0.06	0.87	0.59	0.67
d 11 to 20	1.273	1.345	1.301	1.357	0.06	0.88	0.29	0.74
d 21 to 30	1.835	1.818	1.762	1.818	0.10	0.73	0.85	0.73
d 31 to 41	1.820	1.673	1.932	1.707	0.09	0.68	0.06	0.45
d 1 to 41	1711	1.783	1.727	1.683	0.07	0.51	0.79	0.25

^1^ DWG: daily weight gain; DFI: daily feed consumption; FCR: feed conversion ratio. ^2^ The factorial design (2 × 2) included no aflatoxin or aflatoxin (Afla0 and Afla500 with 0 or 500 µg/kg of aflatoxin/kg of concentrate, respectively) and also included no VB or VB (VB0 and VB800 with 0 or 800 mg of VB/kg of concentrate, respectively). ^3^ Afla: aflatoxin; VB: biocholine. ^a,b^ Differences (*p* ≤ 0.05) between treatments (lines).

**Table 3:** Serum biochemistry of pigs fed with diets containing aflatoxins.

	Treatments ^2^				SEM	*p*-Values ^3^	
Variables ^1^	Afla0VB0 (*n* = 6)	Afla500VB0 (*n* = 6)	Afla0VB800 (*n* = 6)	Afla500VB800 (*n* = 6)		Afla×	VB×
						Day	Day
ALT (U/L)						<0.01	0.15
d 1	17.45	19.36	17.38	18.95	1.61		
d 10	21.26	21.57	21.45	21.39	1.62		
d 20	26.76 ^b^	35.74 ^a^	33.45	29.05	1.62		
d 30	27.43 ^b^	42.57 ^a^	37.95	32.05	1.62		
d 40	32.26	34.24	36.45	30.05	1.62		
AST (U/L)						<0.01	0.23
d 1	38.14	35.74	36.45	38.10	2.47		
d 10	44.12	43.54	44.22	43.45	2.66		
d 20	39.96 ^b^	56.38 ^a^	47.72	48.61	2.66		
d 30	41.96 ^b^	64.88 ^a^	58.72	48.11	2.66		
d 40	41.79 ^b^	53.21 ^a^	49.22	45.78	2.66		
Total protein (mg/dL)						0.87	0.02
d 1	5.10	4.72	5.29	4.23	0.33		
d 10	5.08	4.70	5.58 ^a^	4.20 ^b^	0.33		
d 20	6.44	6.07	6.39	6.13	0.24		
d 30	6.03	5.88	5.86	6.06	0.24		
d 40	6.22	6.28	6.27	6.23	0.24		
Albumin (mg/dL)						0.28	0.38
d 1	3.00	2.82	2.77	3.05	0.18		
d 10	2.99	2.83	2.78	3.05	0.18		
d 20	2.80	2.98	2.86	2.92	0.13		
d 30	3.13	3.43	3.41	3.16	0.13		
d 40	2.71	3.05	2.88	2.88	0.13		
Globulin (mg/dL)						0.95	<0.01
d 1	2.03	1.83	1.83	1.02	0.36		
d 10	2.01	1.82	2.83 ^a^	1.01 ^b^	0.35		
d 20	3.64	3.09	3.53	3.20	0.26		
d 30	2.90	2.45	2.45	2.90	0.26		
d 40	3.51	3.23	3.39	3.35	0.26		
Cholesterol (mg/dL)						0.55	0.65
d 1	66.32	72.81	66.84	72.29	4.80		
d 10	66.44	72.58	66.90	72.12	4.77		
d 20	57.75	57.92	56.92	58.75	3.46		
d 30	56.92	53.00	55.93	54.00	3.46		
d 40	68.00	67.75	70.58	65.17	3.46		
Triglycerides (mg/dL)						<0.01	<0.01
d 1	150.98	105.27	151.35	103.90	17.81		
d 10	200.98 ^a^	105.27 ^b^	202.35 ^a^	103.90 ^b^	17.81		
d 20	48.39	54.44	52.93	59.90	17.81		
d 30	66.23	111.27	65.93	111.56	17.81		
d 40	56.06	55.11	56.93	54.23	17.81		

^1^ ALT: alanine aminotransferase; AST: aspartate aminotransferase. ^2^ The factorial design (2 × 2) included no aflatoxin or aflatoxin (Afla0 and Afla500 with 0 or 500 µg/kg of aflatoxin/kg of concentrate, respectively) and also included no VB or VB (VB0 and VB800 with 0 or 800 mg of VB/kg of concentrate, respectively). ^3^ Afla: aflatoxin; VB: biocholine. ^a,b^ Differences (*p* ≤ 0.05) between treatments (lines).

**Table 4:** Hemogram of pigs fed with diets containing aflatoxins and biocholine (VB).

	Treatments ^1^				SEM	*p*-Values ^2^	
Variables	Afla0VB0 (*n* = 6)	Afla500VB0 (*n* = 6)	Afla0VB800 (*n* = 6)	Afla500VB800 (*n* = 6)		Afla×	VB×
						Day	Day
Erythrocytes (×10^6^ µL)						0.77	0.82
d 1	6.68	6.8	6.68	6.8	0.39		
d 10	6.67	6.78	6.67	6.78	0.39		
d 20	6.019	6.62	6.019	6.62	0.27		
d 30	5.49	5.52	5.49	5.52	0.27		
d 40	6.37	6.26	6.37	6.26	0.27		
Hematocrit (%)						<0.01	0.84
d 1	38.02	37.91	39.01	38.37	1.26		
d 10	38.74	36.51	38.74	36.51	1.25		
d 20	36.41 ^b^	41.43 ^a^	36.41 ^b^	41.43 ^a^	1.25		
d 30	36.91 ^b^	43.59 ^a^	36.91 ^b^	43.59 ^a^	1.25		
d 40	33.69	34.48	33.69	34.48	1.25		
Hemoglobin (g/dL)						0.52	0.96
d 1	10.7	10.64	10.7	10.64	0.47		
d 10	10.76	10.6	10.76	10.6	0.46		
d 20	8.46	8.8	8.46	8.8	0.33		
d 30	7.81	8.13	7.81	8.13	0.33		
d 40	13.21	14.27	13.21	14.27	0.33		
Leukocytes (×10^3^/µL)						0.52	0.96
d 1	10.7	10.64	10.7	10.64	0.47		
d 10	10.76	10.6	10.76	10.6	0.46		
d 20	8.46	8.8	8.46	8.8	0.33		
d 30	7.81	8.13	7.81	8.13	0.33		
d 40	13.21	14.27	13.21	14.27	0.33		
Neutrophils (×10^3^/µL)						0.05	0.60
d 1	5.32	4.99	5.32	4.99	0.56		
d 10	5.3	4.96	5.3	4.96	0.55		
d 20	5.72 ^a^	4.61 ^b^	5.72 ^a^	4.61 ^b^	0.4		
d 30	5.03 ^a^	3.81 ^b^	5.03 ^a^	3.81 ^b^	0.4		
d 40	4.82	5.5	4.82	5.5	0.4		
Lymphocytes (×10^3^/µL)						0.33	0.69
d 1	4.11	4.71	4.11	4.71	0.65		
d 10	4.06	4.62	4.06	4.62	0.64		
d 20	8.36	7.04	8.36	7.04	0.46		
d 30	7.07	6.77	7.07	6.77	0.46		
d 40	4.82	4.76	4.82	4.76	0.46		
Monocytes (×10^3^/µL)						<0.01	<0.01
d 1	0.22	0.18	0.22	0.18	0.03		
d 10	0.25 ^a^	0.18 ^b^	0.25 ^a^	0.18 ^b^	0.03		
d 20	0.23	0.29	0.23	0.29	0.03		
d 30	0.13	0.17	0.13	0.17	0.03		
d 40	0.08 ^b^	0.14 ^a^	0.08 ^b^	0.14 ^a^	0.03		
Eosinophils (×10^3^/µL)						0.12	0.27
d 1	0.32	0.28	0.31	0.29	0.05		
d 10	0.24	0.25	0.24	0.25	0.05		
d 20	0.33	0.46	0.33	0.46	0.05		
d 30	0.37	0.24	0.37	0.24	0.05		
d 40	0.29	0.34	0.29	0.34	0.05		

^1^ In the factorial design (2 × 2), no aflatoxin or aflatoxin (Afla0 and Afla500 with 0 or 500 µg/kg of aflatoxin/kg of concentrate, respectively) and no VB or VB (VB0 and VB800 with 0 or 800 mg of VB/kg of concentrate, respectively) were included. ^2^ Afla: aflatoxin; VB: biocholine. ^a,b^ Differences (*p* ≤ 0.05) between treatments (lines).

**Table 5:** Serum or blood antioxidant response of pigs fed with diets containing aflatoxins and biocholine (VB).

	Treatments ^2^				SEM	*p*-Values ^3^	
Variables ^1^	Afla0VB0 (*n* = 6)	Afla500VB0 (*n* = 6)	Afla0VB800 (*n* = 6)	Afla500VB800 (*n* = 6)		Afla×	VB×
						Day	Day
GST in serum (U GST/mg of protein)						0.75	0.81
d 1	79.7	76.4	83.3	72.9	30.4		
d 10	88.4	83.8	86.9	85.3	16.7		
d 20	106.3	110.4	104.5	112.1	15.5		
d 30	115.8	106.2	107.4	114.6	15.5		
d 40	214.3	235.1	235.1	214.3	15.5		
SOD in blood (nmol SOD/mg of protein)						0.76	0.73
d 1	6.52	5.56	6.1	5.97	1.09		
d 10	6.74	5.68	6.09	6.33	1.08		
d 20	6.19	5.02	5.65	5.56	0.69		
d 30	4.71	4.89	5.1	4.51	0.69		
d 40	7.35	7.7	8.4	6.65	0.69		
CAT in blood (nmol CAT/mg of protein)						0.11	0.94
d 1	18.9	12.6	16.2	15.3	2.7		
d 10	15.6	18.8	16.1	18.3	2.69		
d 20	20.2	20.8	19.8	21.2	2.69		
d 30	14.5	17.1	15.3	16.3	2.69		
d 40	14.2	14.9	15.3	13.8	2.69		
NO_x_ in serum (U NOx/mg of protein)						0.13	0.88
d 1	0.59	0.29	0.32	0.57	0.45		
d 10	0.52	0.3	0.42	0.4	0.28		
d 20	0.61	0.34	0.37	0.59	0.28		
d 30	0.76	1.35	1.07	1.05	0.28		
d 40	0.47	0.37	0.61	0.24	0.31		
ROS in serum (U DCF/mg of protein)						0.20	0.16
d 1	276.4	260.9	268.3	269	32.2		
d 10	331.5	335.9	324.8	342.6	20.3		
d 20	258.2	252.5	283.3	227.4	20.3		
d 30	266.2	278.3	250.4	294.1	20.3		
d 40	266.4	295.4	280.5	281.4	20.3		
TBARS in serum (nmol MDA/mL)						0.77	0.11
d 1	20.1	21.3	19.4	22	1.4		
d 10	11.6	10.5	11.3	10.9	1.62		
d 20	13.2	11.3	12.5	12	1.4		
d 30	9.95	9.79	9.31	10.4	1.4		
d 40	9.15	9.27	8.92	11.5	1.4		

^1^ GST: glutathione S-transferase; SOD: superoxide dismutase; CAT; catalase; NOx: nitric oxide; ROS: reactive oxygen species; TBARS: thiobarbituric acid reactive substance. ^2^ The factorial design (2 × 2) included no aflatoxin or aflatoxin (Afla0 and Afla500 with 0 or 500 µg/kg of aflatoxin/kg of concentrate, respectively) and also included no VB or VB (VB0 and VB800 with 0 or 800 mg of VB/kg of concentrate, respectively). ^3^ Afla: aflatoxin; VB: biocholine.

**Table 6:** Liver, spleen, and jejunum antioxidant concentration of pigs fed with diets containing aflatoxins and biocholine (VB).

Variables ^1^	Combined Treatments ^2^				SEM	*p*-Values ^3^		
	Afla0VB0 (*n* = 6)	Afla500VB0 (*n* = 6)	Afla0VB800 (*n* = 6)	Afla500VB800 (*n* = 6)		Afla × VB	Afla *	VB^+^
Liver								
GST (U GST/mg of protein)	2440	1522	2141	2022	292	0.10	0.05	0.75
NOx (U NOx/mg of protein)	0.75 ^a^	0.52 ^b^	0.43 ^b^	0.70 ^a^	0.07	<0.01	0.75	0.32
ROS (U DCF/mg of protein)	647 ^b^	1426 ^a^	594 ^b^	726 ^b^	84.8	<0.01	<0.01	<0.01
TBARS (nmol MDA/mL)	53.0 ^b^	65.2 ^a^	44.1 ^b^	53.2 ^b^	4.31	0.04	0.03	0.03
Spleen								
GST (U GST/mg of protein)	987	942	380	443	115	0.65	0.94	<0.01
NOx (U NOx/mg of protein)	0.59	0.62	0.65	0.59	0.08	0.57	0.77	0.86
ROS (U DCF/mg of protein)	802 ^a^	595 ^b^	772 ^a^	615 ^ab^	65.6	0.05	0.14	0.24
TBARS (nmol MDA/mL)	48.0 ^a^	53.0 ^a^	47.2 ^a^	34.2 ^b^	4.61	0.05	0.41	0.05
Jejunum								
GST (U GST/mg of protein)	292	1029	519	926	156	0.32	<0.01	0.70
NOx (U NOx/mg of protein)	0.36	0.94	0.38	0.74	0.13	0.38	<0.01	0.44
ROS (U DCF/mg of protein)	456	1541	483	115	219	0.33	<0.01	0.39
TBARS (nmol MDA/mL)	16.7	18.1	15.3	16.9	2.01	0.95	0.47	0.56

^1^ GST: glutathione S-transferase; NOx: nitric oxide; ROS: reactive oxygen species; TBARS: thiobarbituric acid reactive substance. ^2^ The factorial design (2 × 2) included no aflatoxin or aflatoxin (Afla0 and Afla500 with 0 or 500 mg of aflatoxin/kg of concentrate, respectively) and also included no VB or VB (VB0 and VB800 with 0 or 800 mg of VB/kg of concentrate, respectively). ^3^ Afla: aflatoxin; VB: biocholine. ^a,b^ Differences (*p* ≤ 0.05) between four treatments referring to interaction Afla versus VB (lines). Note: * Afla0VB0 versus Afla500VB0; ^+^ Afla0VB800 versus Afla500VB800.

**Table 7:** Pearson coefficients correlations ^1,2^ among antioxidant variables in blood, liver, spleen, and jejunum of pigs fed with diets containing aflatoxins and biocholine (VB).

	Variable ^3^	Blood				Liver				Spleen				Jejunum			
		NOx	TBARS	ROS	GST	NOx	TBARS	ROS	GST	NOx	TBARS	ROS	GST	NOx	TBARS	ROS	GST
Serum	NOx	-	0.30	0.10	0.35 **	−0.33 **	−0.19	−0.22	−0.16	0.30	0.15	−0.10	−0.26	−0.14	−0.01	0.02	0.19
	TBARS		-	−0.03	0.59 *	−0.36 **	−0.09	−0.20	0.60 *	0.05	−0.24	−0.10	−0.30	−0.26	−0.41 *	−0.13	−0.08
	ROS			-	0.37 **	−0.10	−0.23	−0.41 *	−0.10	−0.18	−0.40 *	−0.04	−0.07	−0.14	−0.03	−0.12	−0.05
	GST				-	−0.40 *	−0.15	−0.22	0.18	0.04	−0.43 *	−0.37 **	−0.27	−0.10	−0.25	0.12	0.22
Liver	NOx					-	0.22	−0.06	0.02	−0.16	0.01	0.52 *	0.02	−0.07	−0.01	−0.07	−0.06
	TBARS						-	0.59 *	−0.10	0.21	0.19	−0.06	0.08	0.55 *	0.09	0.38 **	0.25
	ROS							-	−0.25	0.13	0.33	−0.10	0.23	0.50 *	0.01	0.52 *	0.40 *
	GST								-	−0.11	−0.28	0.19	0.01	−0.28	−0.33	−0.34 **	−0.35 *
Spleen	NOx									-	0.11	−0.10	−0.24	0.28	−0.16	0.03	−0.03
	TBARS										-	0.29	0.20	−0.07	0.19	0.08	0.08
	ROS											-	0.33	−0.19	0.04	0.02	−0.02
	GST												-	0.11	0.41 *	0.13	−0.01
Jejunum	NOx													-	0.28	0.46 *	0.36 *
	TBARS														-	−0.01	0.02
	ROS															-	0.94

^1^ Upper row = correlation coefficients (** tendency to differ (*p* ≤ 0.10) and * differing (*p* ≤ 0.05)); lower row, between parenthesis = *p*-values. ^2^ *n* = 24. ^3^ GST: glutathione S-transferase (U GST/mg of protein); NOx: nitric oxide (U NOx/mg of protein); ROS: reactive oxygen species (U DCF/mg of protein); TBARS: thiobarbituric acid reactive substance (nmol MDA/mL).

**Table 8:** Carcass yield, liver weight percentage compared to body weight, spleen weight percentage related to body weight, and jejunum morphometry of pigs exposed to aflatoxin and supplemented with vegetable and biocholine (VB).

Variables	Treatments ^1^				SEM	*p*-Values ^2^		
	Afla0VB0 (*n* = 6)	Afla500VB0 (*n* = 6)	Afla0VB800 (*n* = 6)	Afla500VB800 (*n* = 6)		Afla ×	Afla	VB
						VB		
Carcass yield (%)	68.2 ^c^	69.8 ^bc^	71.1 ^a^	70.6 ^ab^	1.36	0.002	0.695	0.847
% liver weight by body weight	2.36 ^b^	2.57 ^a^	2.37 ^b^	2.43 ^ab^	0.03	0.024	0.001	0.265
% spleen weight by body weight	0.232 ^b^	0.221 ^b^	0.258 ^a^	0.223 ^b^	0.01	<0.001	0.907	<0.001
Jejunum fold (µm)	1642.8 ^a^	1281.9 ^b^	1206.6 ^b^	1523.6 ^a^	67.7	<0.001	<0.001	<0.001
Jejunum villi (µm)	296.1 ^bc^	326.0 ^a^	274.0 ^c^	312.2 ^ab^	14	0.032	0.046	0.552
Jejunum crypt (µm)	223.1 ^b^	256.1 ^a^	225.6 ^b^	250.0 ^a^	11.4	0.02	0.025	0.856

^1^ In a factorial design (2 × 2), no aflatoxin or aflatoxin (Afla0 and Afla500 with 0 or 500 µg/kg of aflatoxin/kg of concentrate, respectively) and no VB or VB (VB0 and VB800 for 0 or 800 mg of VB/kg of concentrate, respectively) were included. ^2^ Afla: aflatoxin; VB: biocholine Different letters (^a–c^ *p* ≤ 0.05) showed difference between treatments (lines).

## Data Availability

Raw data are held by the authors and may be available upon request.

## Appendix A

**Table A1:** The electrospray ionization and MS/MS conditions that were used to determine aflatoxin levels in diets.

Analyte	MRM Transition	Dwell Time (s)	Cone Voltage (V)	Collision Energy (eV)
Aflatoxin B1	313.2 > 285.2	0.01	50	23
	313.2 > 241.2			40
Aflatoxin B2	315.2 > 287.2	0.01	50	26
	315.2 > 259.2			28
Aflatoxin G1	329.2 > 243.2	0.01	40	25
	392.2 > 283.2			25
Aflatoxin G2	331.2 > 245.2	0.01	45	30
	331.2 > 257.2			30
